# Tyrosine Kinase Inhibitors Reduce NMDA NR1 Subunit Expression, Nuclear Translocation, and Behavioral Pain Measures in Experimental Arthritis

**DOI:** 10.3389/fphys.2020.00440

**Published:** 2020-05-27

**Authors:** Karin N. Westlund, Ying Lu, Liping Zhang, Todd C. Pappas, Wen-Ru Zhang, Giulio Taglialatela, Sabrina L. McIlwrath, Terry A. McNearney

**Affiliations:** ^1^Research Division, New Mexico VA Health Care System, Albuquerque, NM, United States; ^2^Anesthesiology, University of New Mexico Health Sciences Center, Albuquerque, NM, United States; ^3^Neuroscience and Cell Biology, University of Texas Medical Branch at Galveston, Galveston, TX, United States; ^4^Neurology, University of Texas Medical Branch at Galveston, Galveston, TX, United States; ^5^Microbiology and Immunology, University of Texas Medical Branch at Galveston, Galveston, TX, United States; ^6^Internal Medicine, University of Texas Medical Branch at Galveston, Galveston, TX, United States

**Keywords:** pain, membrane trafficking, inflammation, glutamate, central sensitization, genistein

## Abstract

In the lumbar spinal cord dorsal horn, release of afferent nerve glutamate activates the neurons that relay information about injury pain. Here, we examined the effects of protein tyrosine kinase (PTK) inhibition on NMDA receptor NR1 subunit protein expression and subcellular localization in an acute experimental arthritis model. PTK inhibitors genistein and lavendustin A reduced cellular histological translocation of NMDA NR1 in the spinal cord occurring after the inflammatory insult and the nociceptive behavioral responses to heat. The PTK inhibitors were administered into lumbar spinal cord by microdialysis, and secondary heat hyperalgesia was determined using the Hargreaves test. NMDA NR1 cellular protein expression and nuclear translocation were determined by immunocytochemical localization with light and electron microscopy, as well as with Western blot analysis utilizing both C- and N-terminal antibodies. Genistein and lavendustin A (but not inactive lavendustin B or diadzein) effectively reduced (i) pain related behavior, (ii) NMDA NR1 subunit expression increases in spinal cord, and (iii) the shift of NR1 from a cell membrane to a nuclear localization. Genistein pre-treatment reduced these events that occur *in vivo* within 4 h after inflammatory insult to the knee joint with kaolin and carrageenan (k/c). Cycloheximide reduced glutamate activated upregulation of NR1 content confirming synthesis of new protein in response to the inflammatory insult. In addition to this *in vivo* data, genistein or staurosporin inhibited upregulation of NMDA NR1 protein and nuclear translocation *in vitro* after treatment of human neuroblastoma clonal cell cultures (SH-SY5Y) with glutamate or NMDA (4 h). These studies provide evidence that inflammatory activation of peripheral nerves initiates increase in NMDA NR1 in the spinal cord coincident with development of pain related behaviors through glutamate non-receptor, PTK dependent cascades.

## Introduction

A critical and unresolved issue in activation dependent neuronal signaling is identification of the numerous signaling molecules and matching them with their specific roles. Discovery and systematic dissection of signaling networks that contribute to inflammatory pain have revealed complex interactions (Hammaker et al., 2003; Kawasaki et al., 2004; Latremoliere and Woolf, 2009), initiated after the significant rise of glutamate following an inflammatory insult (Sluka and Westlund, 1992, 1993a,b; Sorkin et al., 1992). Non-receptor Src family protein tyrosine kinase (PTK) interactions with tyrosine residues of ionotropic NMDA receptor NR2A/B subunits impact phosphorylation, channel protein insertion, and activation state, particularly in the hippocampus as it impacts long term potentiation (LTP). The NMDA receptor NR1 subunit, part of this heteromeric ion channel, has been less well studied since it was reported that immunoprecipitated NR1 is not phosphorylated on tyrosine residues in hippocampal preparations (Lau and Huganir, 1995; Huang and Hsu, 1999).

In the present study, NR1 expression increase in the lumbar spinal cord was initiated by knee joint inflammation with k/c injection. Two PTK inhibitors (genistein and lavendustin A) and their inactive analogs (diadzein and lavendustin B) were investigated to determine their influence on pain related behavior, spinal cord content and cellular localization of NR1 after inflammatory hindlimb insult. The PTK inhibitors reduced spinal cord NR1 neuronal expression and nuclear translocation in the spinal cord, as well as improved secondary heat hypersensitivity on the footpad in an experimental knee joint arthritis.

## Materials and Methods

### Animals

All studies followed the Institutional Animal Care and Use Committee Guidelines, in accordance with the guidelines of the National Institutes of Health and the international Association for the Study of Pain. Eighty-eight Sprague−Dawley rats (250−300 g, Harlan) were used in these pharmacologic, biochemical, behavioral and immunocytochemistry studies. All efforts were made to minimize animal suffering, to reduce the number of animals used, and to utilize alternatives to *in vivo* techniques, when available. All animals were housed in a room with a constant ambient temperature of 22°C and 12 h light/dark cycle with free access to food and water.

### Pre-treatment in Rats With Acute Monoarthritis

On Day 1, anesthetized animals received surgical implantation of a microdialysis fiber for spinal administration of PTK inhibitors and inactive analogs. On Day 2, baseline behavioral testing was followed by pre-treatment infusion of agents for 1.5 h, prior to induction of knee joint inflammation under brief anesthesia. Behavioral testing was repeated 4 h after induction of joint inflammation. Use of a k/c knee joint injection (k/c, 3%/3%, 0.1 ml in saline) acute inflammatory pain model allows clear separation of the zone of inflammation and the sensitized hindpaw for testing responses (secondary hyperalgesia) indicative of central sensitization. Animals were anesthetized and either (i) transcardially perfused with paraformaldehyde (PFA) for light and EM immunohistochemical and immunogold studies, or (ii) fresh, frozen tissues collected for biochemical analysis.

### PTK Inhibitors and Cycloheximide

Two PTK inhibitors and their inactive analogs were compared in these studies (*n* = 30). Genistein (5, 7-dihydroxy-3-(4-hydroxyphenyl)-4H-1-benzopyran-4-one; 4’,5,7- trihydroxy-isoflavone, Cat # G-103. RBI, Natick, MA, United States) is a reversible PTK inhibitor that decreases NMDA currents in hippocampal patch clamp studies (Akiyama et al., 1987; Wang and Salter, 1994; Yu et al., 2016). Daidzein (4’,7- Dihydroxyisoflavone), is an analog of genistein that lacks PTK inhibitory activity and had no effect on NMDA currents in patch clamp studies. Lavendustin A (5-Amino-[N-2,5-dihydroxybenzyl-N’-2- hydroxybenzyl] salicylic acid, Cat # 428150, Lot #B42143 Calbiochem, LaJolla, CA, United States), is a structurally distinct PTK inhibitor that reversibly depressed NMDA currents, and was tested along with its inactive analog, lavendustin B (5-amino-(N,N’-*bis*-2-hydroxybenzyl) salicylic acid, Cat # 428160, Lot # B39919 & B49301, Calbiochem). The drugs (genistein, lavendustin A & B and daidzein) were dissolved in the 50% dimethyl sulfoxide (DMSO) in aCSF vehicle.

Cycloheximide is a widely used protein synthesis inhibitor and actinomycin D, a potent transcription inhibitor. Cycloheximide [*n* = 3, 30 mg/kg, intraperitoneal (i.p.)] or actinomycin D (*n* = 3, 10 mg/kg, i.p.) were given in some *in vivo* studies to determine if the observed staining density and intracellular localization changes were due to *de novo* NR1 protein synthesis.

### Spinal Microdialysis Installation for Drug Infusion

One day prior to the induction of arthritis, the microdialysis fiber was surgically implanted in anesthetized rats (sodium pentobarbital, 40 mg/kg) (Skilling et al., 1988; Sluka and Westlund, 1992). A small midline incision was made in the skin over the L1 vertebral level. The vertebrae were cleared of muscle and 1 mm holes were drilled in the lateral aspect on both sides of the L1 vertebrae to expose access to the L3-L4 spinal segment. A microdialysis fiber (200 μm o.d., 45,000 MW cut-off, Hospal, AN69) was threaded transversely across the dorsal horn through the holes and stabilized with dental cement. The microdialysis fiber was coated with epoxy except for the 2 mm permeable portion passing through the spinal gray matter. The microdialysis fiber was connected to PE_20_ tubing (Becton Dickinson & Co., San Diego, CA, United States) which was tunneled under the skin to the nape of the neck. In surgical control animals, the microdialysis fiber was implanted only in the subcutaneous tissue over the back muscle and similarly tunneled to the neck. This facilitated the blinded approach. The aCSF was infused (5 μl/min) for 1.5 h and then the tube was heat sealed for use on the following day.

### Dose Response and Estimate of Drug Delivered

Based on estimates for genistein concentrations measured by spectrophotometer (Beckman DU650, Beckman Coulter, Inc., Fullerton, CA, United States) after transfer across the microdialysis membrane into aCSF, a maximum of 5.6% of the drug dose in the tube crosses into the tissue. Therefore, with the diffusion barriers presented by the tissue, the neurons are likely to have been maximally exposed to <56 μM genistein or <25 μM lavendustin, much lower than the 1 mM concentration inside the microdialysis fiber. It has been shown that doses of genistein greater than 30 μM can directly inhibit NMDA channel activation, while lower doses and lavendustin A inhibit PTK activation and have no direct effect on NMDA channels (Huang et al., 2010).

Dose response curves for paw withdrawal latency in response to stimulation with heat were generated for animals receiving genistein spinally at concentrations inside the microdialysis of 200 μM (*n* = 3), 500 μM (*n* = 4), 1 mM (*n* = 9), and 2 mM (*n* = 3). The most effective pre-treatment dose for spinally administered genistein was 1 mM. Animals received spinal lavendustin A or B as positive and negative controls for genistein, respectively. Concentrations tested were 0.1 mM (*n* = 3), 0.5 mM (*n* = 3), and 1 mM (*n* = 5). The most effective dose of lavendustin A in these pre-treatment studies was 0.5 mM. A genistein dose of 1 mM was used for the surgical control animals (*n* = 3) receiving superficial implantation of the microdialysis tubing for subcutaneous drug delivery. Daidzein was also tested at a dose of 1 mM (*n* = 3). Also, eight additional control animals received the vehicle (V), 50% DMSO in aCSF.

### Induction of the Knee Joint Inflammation Model

An acute inflammatory response restricted to the knee joint was induced when 3% kaolin and 3% carrageenan (k/c; in sterile saline; 0.1 ml; pH 7.4) was injected directly into the joint cavity while animals were briefly anesthetized with sodium methohexital (Brevital, 40 mg/kg, i.p., Mettawa, IL, United States). The knee joint was flexed manually until the rat awakened (approximately 5-10 min). Behavioral testing was initiated 4 h after k/c intra-articular injection. In this k/c arthritis model, localized joint swelling, as well as limping and guarding of the limb, were well developed 4 h post-injection (Sluka and Westlund, 1993a). Surface temperature readings of both knees were obtained with a digital infrared temperature probe. Bilateral knee joint circumferences were obtained with a thin, flexible tape measure around the center of the knee joint with the limb held in extension. Measurements were collected before (baseline) and 4 h after intra-articular injection of the knee joint with k/c after the rat was re-anesthetized with sodium pentobarbital (40 mg/kg, i.p), just prior to perfusion with 4% paraformaldehyde (PFA).

### Behavioral Assessment

Fifty-eight rats were used for the behavioral studies. The nociceptive behavioral measure documented bilaterally was decreased threshold withdrawal response to radiant heat, reflecting secondary heat hypersensitivity and central sensitization (Hargreaves et al., 1988; Sluka et al., 1994). Animals were placed in Lucite cubicles on a glass top table cooled with a fan and allowed to accommodate for 30 minutes prior to testing. A hand-held metal box focusing a high intensity light through an aperture (1 × 0.8 cm) was used to apply radiant heat through the glass to the plantar surface of the hindpaw until the animal lifted its hindpaw. The time delay until reflexive withdrawal response was considered the paw withdrawal latency (PWL), measured in seconds (s). Both hindpaws were tested independently at 5 min intervals for a total of 5 trials. A mean of these 5 readings was used as PWL response at each time point. The same observer performed the tests for each group under blinded conditions. A 15 s duration was used as a maximum cutoff, but was never employed. In the experimental rats, the PWL was measured before (baseline) placement of the microdialysis fiber (Day 1) and after administration of drug or vehicle had been infused for 1.5 h (Day 2) at which time k/c was injected into the knee joint. The final measurement for PWL was 4 h after induction of arthritis.

### Immunocytochemical Localization of NMDA Receptor Subunit NR1

#### Animal Preparations

Animals were anesthetized and transcardially perfused with a brief warm saline rinse followed by fixative solution (4% PFA in 0.1 M phosphate buffer, pH 7.4). The lumbar spinal cord (L4-6) was dissected and tissues were soaked overnight in 30% buffered sucrose and cut frozen at 30 μm on a sliding microtome. Tissue sections were randomly selected from serial sets spaced at least 100 μm apart. The tissues from some animals (*n* = 8) were briefly permeabilized with a 50% buffered ethanol treatment or Triton-X 100 added to the diluent.

#### Spinal Cord Immunohistochemistry for NMDA NR1 Subunit

Tissue sections from twenty one rats were stained immunocytochemically for glutamate receptors NR1, NR2A/B, NR2C, mGluR1 and mGluR5 using commercial antibodies (0.5−1 μg/ml, Chemicon International, Temecula, CA, United States). None of these receptors had staining associated with the nucleus in unstimulated conditions. The NR1 antibody was validated with RT-PCR and immunoprecipitation (McNearney et al., 2010). The primary antibodies were diluted in phosphate buffered saline (PBS) with 0.1% BSA and 0.1% Triton-X-100 for overnight incubation on the spinal cord tissues at room temperature on a rotating shaker. After washing in PBS (pH 7.6), the tissue sections were incubated in the appropriate secondary antibody, either anti-mouse or anti-rabbit IgG (1:200, 90 min). Sections were reacted with DAB (1.5 mg/ml) solution as the chromogen and peroxide (3%, 0.5 μl/ml) to produce a brown reaction product. Semi-quantitative staining density measure was determined using NIH ImageJ (*n* = 5).

#### Spinal Cord Immunohistochemistry for PhosphoNR1 in a Rat Model of Chronic Trigeminal Nerve Pain

To validate the nuclear immunostaining, confirmation was sought in another chronic pain model, the chronic constriction injury of the infraorbital nerve (CCI-ION) (Vos et al., 2000). The unilateral chronic constriction injury (CCI) was induced on the infraorbital trigeminal nerve (ION) branch. The rat was anesthetized with isoflurane (4.0% vol in 1.0 l/min oxygen), and the left ION dissected free within the orbital cavity. Two chromic gut sutures (5-0, Ethicon 634G, Ethicon, Somerville, NJ, United States) were tied loosely around the left ION (2 mm apart) while the naive control animal did not undergo surgery or anesthesia (*n* = 3). The incision was closed using 5-0 nylon suture (Cat. # MV-661, Med-Vet International, Mettawa, IL, United States) and animals recovered in less than 15 min following surgery. Mechanical sensitivity was tested on the whiskerpad at baseline prior to CCI surgery and weekly for the following 12 weeks when animals were euthanized, transcardially perfused with 4% PFA, and cervical spinal cord excised, cryoprotected in 30% sucrose, embedded in Optimal Cutting Temperature compound (OCT, Thermo Fisher Scientific, Ottawa, Ontario, Canada) and frozen at −80°C.

Tissue sections were cut at 20 μm thickness and collected on glass slides, washed with PBS, blocked (3% normal goat serum, 0.01% Triton X-100, in PBS) and incubated overnight in anti phosphoNR1 antibody (1:2,000; Upstate, New York, NY, United States) at room temperature on a rotating shaker. Antibody staining was visualized using a goat anti-rabbit secondary antibody conjugated to Alexa Fluor 488 (Molecular Probes) and counterstained with the nuclear dye DAPI. Images were taken at 100x magnification using light optics on the Fluoview FV1200 confocal microscope (Olympus, Center Valley, PA, United States).

#### Immunocytochemical Method Controls

Immunocytochemical method controls included sections processed in the absence of the primary or secondary antibody or Vectastain avidin-biotin complex (ABC) kit reagents (1 h, Vector Laboratories, Burlingame, CA, United States). The specificity of the transmitter antibodies was confirmed by appearance of a single band by Western blot using the C-terminus NR1 antibody from (0.5 to 1 μg/ml; Chemicon, Cat #AB1516, Temecula, CA, United States). This antibody identified a splice variant containing a nuclear localization sequence region. An N-terminus NR1 antibody (Upstate, New York, NY, United States) provided equivalent localization in spinal cord and in cultured human clonal SH-SY5Y cells. To further intensify the reaction product, particularly for visualization of nuclear rings, some of the tissues were treated with colloidal gold conjugated IgG and reacted with a silver chloride solution to produce an intense black reaction product.

As a positive control, immunocytochemical staining for NFκB p50 was also performed, with diluent only as negative control. Inhibition of nuclear localization with tyrosine kinase inhibitor genistein was demonstrated in our tandem controls, as has been previously reported for IκB p50 subunit degradation and NFκB nuclear localization (Garcia Palacios et al., 2005). Increased staining for NR1 and nuclear translocation were also inhibited by NMDA receptor antagonist, MK-801, as a negative control in cultured cells.

#### Electron Microscopy

Ultrastructural analysis by EM was performed on spinal cords with pre- and post-embedding immunostaining. Two groups of rats (*n* = 3 each, control and 4 h after k/c induced arthritis) were deeply anesthetized, then perfused via ascending aorta puncture with a mixture of 2.5% glutaraldehyde and 1% PFA. The lumbar cords were removed and cut at a 30 μm thickness with a vibratome. The spinal cord sections were incubated overnight in 1% sodium borohydride, then 50% buffered ethanol (pH 7.4).

##### Pre-embedding immunostaining

Tissue sections were blocked and incubated with mouse anti-NMDA NR1 subunit monoclonal antibody overnight (Pharmingen, San Diego, CA, United States). After washing with PBS (pH 7.6, ×6), tissue sections were incubated in secondary antibody, biotinylated horse anti-mouse IgG (1:100) for 2 hr. NMDA NR1 subunit was visualized after incubation with ABC kit reagents (1 h) and reaction with the chromogen DAB (1.5 mg/ml) and peroxide (3%, 0.5 μl/ml) to produce an electron dense product (Hsu et al., 1981). Final sections were treated with osmium, dehydrated through graded alcohols and embedded in Poly/BED 812 resin for visualization with an electron microscope (JEM-100CX, JEOL, Ltd., Tokyo, Japan).

##### Retrogradely labeled spinothalamic tract neurons

In 4 animals, injections were made into the ventral posterolateral thalamus with wheat germ agglutinin conjugated with horseradish peroxidase (WGA- HRP, Sigma-Aldrich, St. Louis, MO, United States) to identify spinal projection neurons prior to processing for immunocytochemical staining (Ye and Westlund, 1996). The WGA-HRP was visualized as large, dense crystals using tetramethybenzidine stabilized with ammonium paratungstate and DAB. The crystals were clearly differentiated from the amorphous immunohistochemical staining for the NMDA NR1 subunit.

##### Post-embedding colloidal gold immunohistochemistry

With post-embedding immunogold histochemistry the antibody was bound to small round gold beads to identify the NMDA NR1 subunit (Westlund et al., 1992). Ultrathin sections on single-slot formvar coated nickel grids were etched sequentially in 1% solution of periodic acid for 10 min, 20% sodium meta-periodate for 15 min and 1% sodium borohydrate for 10 min. The sections were washed in distilled water between solutions in 1:30 dilution for 30 min. Sections were placed on droplets containing the mouse anti-NMDA NR1 antibody (1:100; Pharmingen) overnight at 4°C. After wash in 0.05 M Tris−buffered saline (TBS) (pH 7.2) with 0.2% bovine serum album (BSA), grids were incubated in the rabbit anti-mouse IgG coupled to 10 nm gold spheres (1.5 h TBS, pH 8.2, Janssen Pharmaceutica, Beerse, Belgium), washed twice in TBS buffer (pH 7.2) then distilled water, prior to uranyl acetate (5 min) and lead citrate (1−1.5 min) for contrast.

#### Immunocytochemistry for Clonal Neuronal Cells

Clonal human SH-SY5Y neuroblastoma cells (CRL2266, ATCC, Bethesda, MD, United States) were plated on poly-D-lysine (PDL) treated glass coverslips and maintained in a humidified atmosphere of 95% air and 5% CO_2_ at 37°C in Neurobasal Medium (Invitrogen, Carlsbad, CA, United States). For nuclear translocation experiments, plated cells were incubated with 100 μM L-glutamate or vehicle for 4 h at 37°C. In some conditions, cells were preincubated with 50 μM genistein or 100 nM staurosporin, a broad spectrum tyrosine kinase inhibitor (Calbiochem) for 5 min at 37°C before the addition of 100 μM glutamate. After the incubation, the cells were fixed with 4% PFA for 1 h room temperature in preparation for staining. Three replicates minimum were studied.

Immunocytochemistry was performed using PBS containing Mg^2 +^ and Ca^2 +^ (PBSAT). All procedures were performed at 4°C. Cells were washed three times and post-fixed in ice-cold 70% methanol for 30 min. Fixed cells were incubated with the mouse anti- NMDA NR1-specific antibody (1:100, Pharmingen) in 1% BSA (Fraction V, Sigma) and 0.025% Tween-20 (1% PBSAT) for 16 h at 4°C on a rotating shaker. After the primary antibody was removed, the cells were washed 3 X in 1% PBSAT, then incubated with rabbit anti-mouse Alexa Fluor 488 green (1:100 dilution, 30 min, Molecular Probes, Inc., Eugene OR, United States). Cells were washed 3 X with 1% PBSAT, glass coverslips mounted on slides with 20% glycerol-PBS. Computer-assisted quantification was performed on images taken with a Nikon FXA confocal laser microscope equipped with a SPOT digital system (Nikon Instruments, Melville, NY, United States).

### Western Blot Analysis Protocols

#### Preparation of Rat Spinal Cord for Western Blot

Rats were anesthetized at experiment end and perfused for 2 min with ice cold saline/heparin. Spinal cord L4-L6 laminae I and II were dissected and homogenized in Radioimmunoprecipitation assay (RIPA, Sigma) lysis buffer with protease inhibitors at 4°C. Samples from treated spinal cord (40 μg protein) and β-actin control (3 μg) were diluted with running buffer and loaded on 8% gels for electrophoresis (100 mv) separation for 2 h, 20 min. Protein was transferred (85−70 mv) for 90 min. The membranes were blocked with 5% milk in Tris−buffered saline with Tween-20 (TBST) for 1 hr prior to overnight incubation in mouse anti-NMDA NR1 antibody (1:1,000 in 2.5% milk TBST, 4°C, Pharmagen). After washing (5 × 6 min), membranes were incubated in goat anti-mouse IgG-HRP in 2.5% milk TBST (1:5,000) for 1 h after extensive wash and enhanced chemiluminescence (ECL) detection. Three replicates minimum were studied.

#### Preparation of Nuclear and Cytosolic Protein Extracts of Cultured Cells

SH-SY5Y cell cultures were plated at 10^7^/treatment group and grown to 80% confluence, then incubated 16 h in serum free F12:DMEM. Subsequently the cells were treated with final NMDA concentrations of 0, 0.1, 1.0, and 10 μM or control (water, 0.001%) in F12:DMEM for 6 h. All protein isolation procedures were performed at 4°C (Nuclei Isolation kit, NUC-101, Sigma). Cells were washed twice with divalent cation-free PBS and detached from tissue culture flasks using 1 mM EDTA in PBS. The cells were pelleted at 500 × g for 5 min and resuspended in 300 μl lysis buffer consisting of 10 mM Tris−HCl (pH 7.4), 2 mM MgCl_2_, protease inhibitors (40 μg/ml phenyl methylsulfonylfluoride, 100 μg/ml leupeptin, 1 μg/ml aprotinin) and 1% thiodiglycol. Cells were lysed with 30 strokes of a type-B pestle and small fractions monitored with a microscope to confirm complete lysis of cells and intact nuclei. A 50 μl aliquot was reserved as total protein extract. The nuclei were pelleted at 800 × *g* and the supernatant (cytosolic fraction) removed and frozen at −80°C. The nuclei were washed once with lysis buffer and placed in 100 μl nuclear extraction buffer (400 mM NaCl, 20 mM HEPES pH 7.9, 1 mM EDTA, 1 mM EGTA, 1 mM dithiothreitol, protease inhibitors), resuspended by vortexing, and placed on a shaker at 1400 RPM in a 4°C room for 20 min. The non-soluble matter was pelleted at 10,000 × *g* for 20 min and the supernatant (nuclear fraction) removed and frozen at −80°C. Three replicates minimum were studied.

#### SDS−PAGE and Immunoblot Assays

For SDS−PAGE, 40 μg of each of the lysates was added to an equal volume of 2 x Laemmli Buffer (62.5 mM Tris−HCl pH 6.8, 2% [wt/vol] SDS, 20% [vol/vol] glycerol, 5% [vol/vol] 2-mercaptoethanol) and heated at 95°C for 5 min. The samples were resolved on pre-cast 7.5% polyacrylamide gels (Bio Rad, Hercules, CA, United States) and transferred to PVDF Hybond-P membranes. Protein was transferred (85−70 mv) for 90 min. The membranes were blocked with 5% milk in Tris−buffered saline with Tween-20 (TBST) Tris−HCL pH 8.3, 39 mM glycine, 20% methanol [vol/vol] at 80 V for 2 h. Primary anti-NMDA NR1 antibodies included a monoclonal recognizing the N-terminus (Pharmingen) and a polyclonal recognizing the C-terminus (1:100, Chemicon). Immunoreactive bands were visualized with enhanced chemiluminesence (Amersham). Three replicates minimum were studied.

### Statistics

Data are presented as mean values plus or minus standard error (SE) Student’s *t*-tests were used to assess significant differences in mean values between paired and unpaired groups in some studies. Comparative analyses among the treatment groups versus the vehicle group were performed on immunohistochemical and behavioral data collected at baseline and after 4 h of inflammation with one–way ANOVA and Newman-Keuls Multiple Comparison (behavior and immunostaining density) or Mann-Whitney *U*-test (*in vitro* study) *post hoc* testing. A *p-*Value < 0.05 was considered significant.

## Results

### Pretreatment With Genistein Attenuated Heat Hypersensitivity in Rats With k/c Induced Arthritis

**FIGURE 1:**
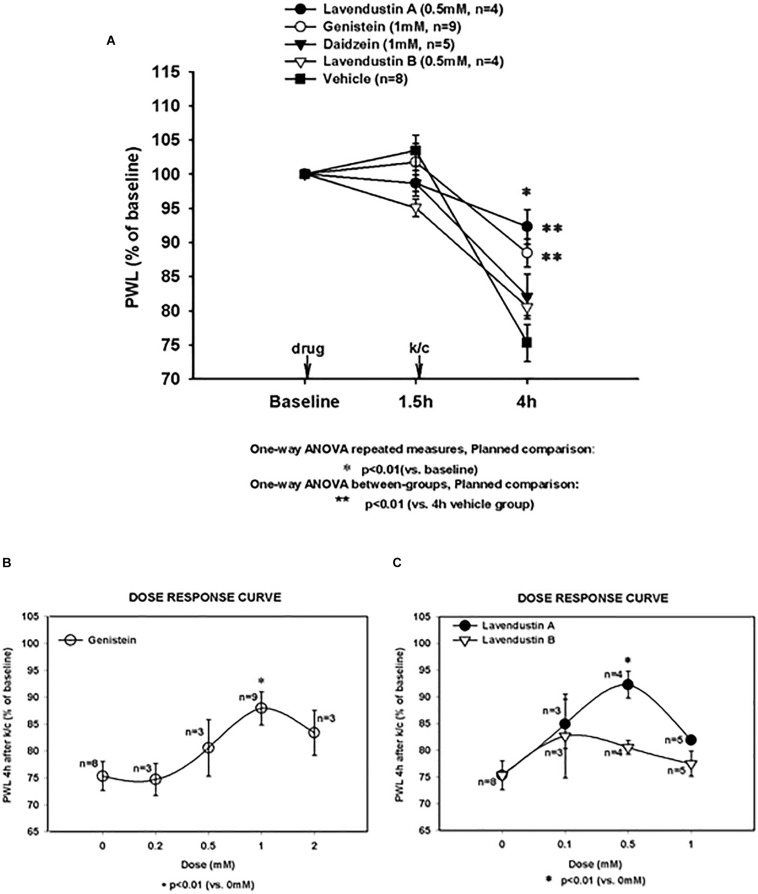
Arthritis induced hypersensitivity (4 h) and effects of non-receptor tyrosine kinase inhibitor pre-treatments. **(A)** Paw withdrawal latency (PWL) decreases induced by knee joint inflammation were significant in all treatment groups (*significantly different from baseline for all groups; *p < 0.01). Pretreatment with genistein (Gen) and lavendustin A (Lav A) significantly (**p < 0.01 versus 4 h vehicle group) attenuated PWL decreases observed in animals with kaolin and carrageenan (k/c) arthritis induction 1.5 h post-drug infusion. Pretreatment with lavendustin B (Lav B) or diadzein (Dia) had no effect. **(B)** Dose response curve is shown for pretreatment with genistein and **(C)** lavendustin A and B. Lav A (*n* = 4), Gen (*n* = 9), Dia (*n* = 5), Lav B (*n* = 4), and Veh (*n* = 8). Dosing was provided intraspinally by microdialysis fiber, *p < 0.01 compared to 0 mM, one way ANOVA repeated measurement, planned comparison.

Paw withdrawal latency (PWL) to radiant heat was significantly reduced from baseline at 4 h after induction of the k/c knee joint inflammation. As we have reported previously in this inflammatory arthritis model in rats, reduced PWL is indicative of central sensitization and secondary heat hyperalgesia (Sluka and Westlund, 1993a). Figure 1 demonstrates behavioral responses in the vehicle and PTK inhibitor analog pretreatment groups with k/c arthritis. The decrease in PWL response occurred maximally on the side ipsilateral to the inflamed knee 4 h in control animals and was linearly correlated with the increase in joint swelling. In the present study, similar responses were demonstrated for arthritic rats intrathecally treated with vehicle (75.3% of baseline, *p* < 0.01).

Pre-treatment with genistein (1 mM) and the other related agents tested in naïve animals did not alter PWL values from baseline PWL, as demonstrated at 1.5 h. However, as shown in Figure 1A, all treatment groups with arthritis had PWL thresholds significantly less than baseline (*p* < 0.01). Pre-treatment with the PTK inhibitor, genistein, significantly attenuated the inflammation-induced decrease in PWL in this arthritis model (88.47% of baseline, *p* < 0.01 compared to vehicle pretreated arthritic rats).

The structurally distinct PTK inhibitor, Lavendustin A, also significantly abrogated the development of secondary hyperalgesia (92.28% of baseline) compared to vehicle. Secondary hyperalgesia typical of this model developed after administration of vehicle and the inactive analogs, daidzein and lavendustin B (75.32, 82.12, and 80.54% of baseline, respectively). Dose response curves are shown for genistein, lavendustin A, and lavendustin B (Figures 1B,C, respectively), and were used to derive the optimal doses for the full study.

### Joint Temperature and Circumference in Arthritic Joints Were Unaffected by Intraspinal Genistein

Surface joint temperature (degrees Celsius) and circumference (cm) were measured at baseline, and at 4 h, when the animals were tested for secondary hyperalgesia. In all arthritic groups, the ipsilateral joint temperatures were significantly elevated compared to baseline levels (105 − 108% increase over baseline, *p* < 0.02). In addition, the ipsilateral joint circumferences were significantly increased compared to baseline levels (114 − 118% over baseline, *p* < 0.04). Although secondary hyperalgesia values were significantly blunted at the 4 h time point with intraspinal genistein and lavendustin A, increases in joint circumferences and surface temperatures in the arthritic joints were similar to the vehicle pretreated arthritic animals.

### Increased Expression of Spinal NMDA NR1 Subunit 4 h After Induction of k/c-Induced Arthritis and Reduction by Genistein

#### NMDA NR1 Expression Changes in Spinal Cord Immunostaining

**FIGURE 2:**
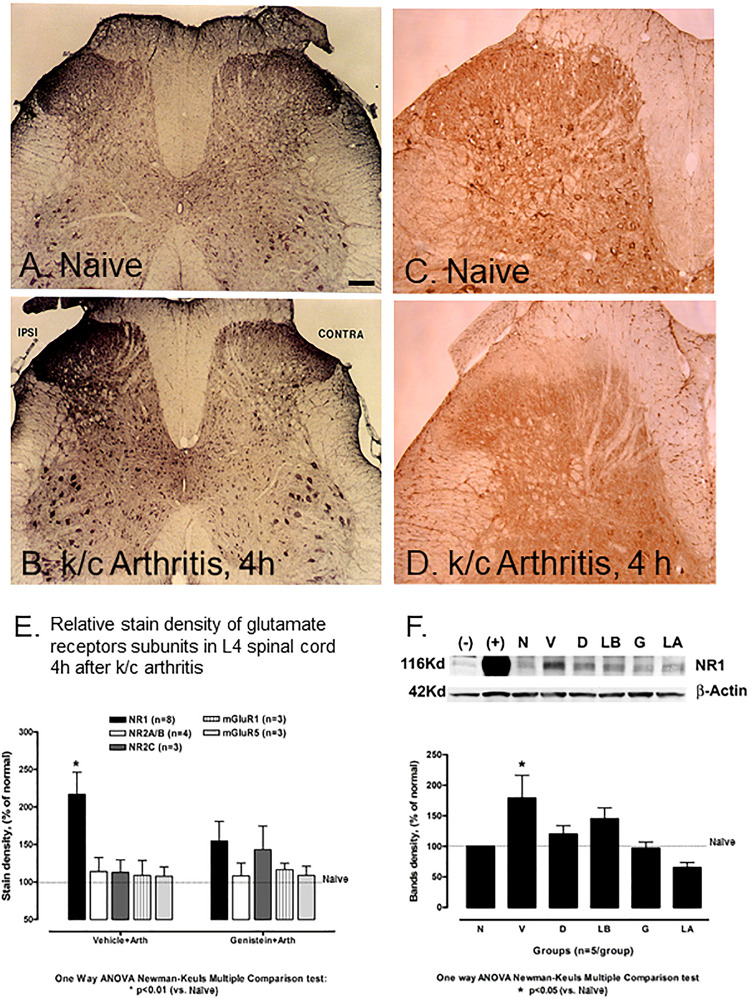
Effect of non-receptor tyrosine kinase inhibitors on NMDA NR1 and immunostaining of NR1, pNR1, and other NMDA receptor subunits in rat L-4 spinal cord. **(A)** Representative photomicrograph of NMDA NR1 intensified immunostaining in a naïve animal. **(B)** NMDA NR1 in an arthritic rat with ipsilateral k/c knee joint inflammation at 4 h. **(C)** PhosphoNR1 immunostaining distributed uniformly in the dorsal horn of naïve rats. **(D)** PhosphoNR1 immunostaining diminished at 4 h, particularly in dorsal horn laminae I and II in rats with k/c arthritis. **(E)** Relative staining density of NMDA NR1 in animals with k/c arthritis (4 h) was significantly increased in the superficial ipsilateral dorsal horn (I-II) compared to naives when vehicle pre-treated, but not when pre-treated with genistein. Staining densities of other subunits tested did not significantly change compared to controls, with or without genistein. (*n* = 3–8) *p < 0.01 compared to the naive group **(F)** NMDA NR1 protein expression band density was increased significantly in spinal cord (L4-6) of animals with k/c arthritis (4 h) determined with Western blot analysis. Pretreatment with genistein, lavendustin A, and related agents successfully prevented the increase. Densities shown in the bar graph were corrected relative to β-actin. Positive (+) and negative (–) controls are provided. N, naïve; V, vehicle; D, daidezein; LB, lavendustin B; G, genistein; LA, lavendustin A pretreatments in arthritic rats. *p < 0.05 compared to the naive group. One way ANOVA with Newman–Keuls Multiple Comparison *post hoc* testing. Scale Bar = 100 μm in panels **(A,B)** and 50 μm in panels **(C,D)**.

While NMDA NR1 immunostaining in the dorsal and ventral horns of the lumbar (L4) spinal cord was abundant in naïve non-arthritic rats (Figure 2A), NMDA NR1 immunostaining was visibly increased in the ipsilateral dorsal horn in rats with knee joint k/c induced monoarthritis at 4 h (Figure 2B). Analysis of immunostaining density demonstrated that NMDA NR1 staining in the lumbar spinal cord lamina I and II ipsilateral to the inflamed knee joint was greatly increased in dorsal horn in vehicle injected arthritic rats compared to naïve animals (216.95 ± 29% vs. 100%, respectively, *p* < 0.01) (Figure 2E). Arthritis induced increase in NMDA NR1 immunostaining was blunted by genistein pretreatment compared to those pretreated with vehicle (154.53 ± 26 vs 216.95 ± 29, respectively, *p* = 0.07), and was not significantly increased compared to naïve controls.

The phosphoNR1 was also identified with immunostaining and noted to be distributed uniformly in the dorsal horn of naive rats (Figure 2C). Immunostaining for phosphoNR1 was visibly diminished at 4 h in rats with k/c arthritis, particularly in dorsal horn laminae I and II (Figure 2D). Similarly in a separate study, at 12 weeks a visible decrease in phosphoNR1 was detected in the cervical spinal cord of a rat model of chronic neuropathic trigeminal pain compared to naïve control (Supplementary Figure S1).

#### Increased Spinal NMDA NR1 Protein Expression in Knee Joint Arthritis Reduced by Pretreatment With Genistein or Lavendustin A Shown by Western Blot

Figure 2F demonstrates with Western blots and bar graphs, the relative densities of band patterns of NMDA NR1 protein lysates derived from the lumbar spinal cord from non-arthritic and arthritic rats (*n* = 5/group). In these studies, compared to naïve non-arthritic rats (N), the NMDA NR1 band densities for spinal cord of arthritic rats (V, vehicle treated) were significantly increased (100 vs. 179 ± 39%, *p* < 0.05).

Groups of rats were also intraspinally pretreated with genistein, with genistein’s active (lavendustin, LA), or with inactive (lavendustin B, LB) or (daidzein, D) analogs. NMDA NR1 band densities were not increased in arthritic rats treated with PTK inhibitor genistein or its active analog lavendustin A, compared to the naive non-arthritic rats (N, 97 ± 10.2 and 65.21 ± 8.4 respectively). Arthritic rats treated with inactive analogs daidzein or lavendustin B had increased band densities over the normal controls but were not significantly different from the vehicle treated arthritic rats (120 ± 13.6 and 145 ± 18, respectively). There were no increases in NMDA NR1 receptor in any group in the contralateral dorsal horn of the spinal cord.

### Other Spinal Glutamate Receptors Were Less Affected or Unchanged in k/c-Induced Arthritis

Other glutamate receptor subunits tested included ionotropic NR2A/B, NR2C, metabotropic mGluR1 and mGluR5. These subunits did not significantly increase in the spinal cord in vehicle treated animals at 4 h after k/c injection (113.98 ± 19, 112.87 ± 16.5, 108.81 ± 19.8 and 107.65 ± 12.31, respectively), (Figure 2E, left bars) nor did they stain the nucleus. NR2A/B, mGluR1 and mGluR5, also did not significantly increase above background values in the arthritic rats pretreated with genistein (108.04 ± 17.5, 116.46 ± 8.72 and 110.97 ± 10.14%, respectively). Ionotropic NMDA NR2C was slightly elevated (Figure 2E, right bars) compared to the naive controls.

Parallel control studies determined that results obtained in animals with k/c induced arthritis in the absence of vehicle pretreatment or with sham surgery alone (external subcutaneous microdialysis fiber) matched those of arthritic animals with vehicle pre-treatment. Neither vehicle treated animals nor those with k/c induced arthritis demonstrated increased staining densities on the contralateral side or compared to naive, non-arthritic controls.

### Nuclear Translocation of NMDA NR1 and Blunting by Genistein

#### NMDA NR1 Immunostaining Decrease by Genistein

**FIGURE 3:**
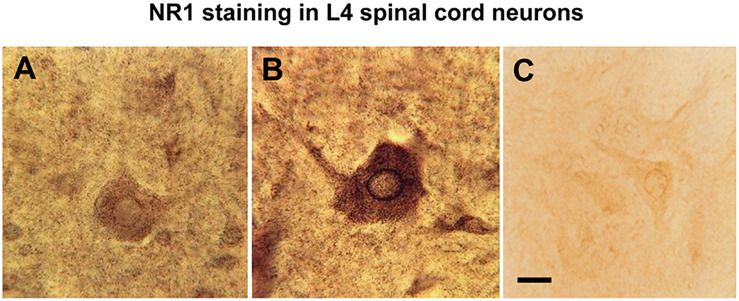
Nuclear translocation of NMDA NR1 in arthritic rats and effect of genistein. **(A)** Representative high power light photomicrograph of NMDA NR1 immunostained spinal neurons in the ipsilateral lumbar cord of a naïve rat. **(B)** In arthritic rats (4 h) with vehicle pretreatment, increased NR1 staining is appreciated visually in the cytoplasm and nuclear rim. **(C)** The staining density of NMDA NR1 was markedly reduced in arthritic rats pretreated with genistein when tissue sections were taken near the microdialysis fiber. (*n* = 3) Scale bar = 25 μm for all photomicrographs.

The rapid increase in NR1 staining within 4 h of k/c joint injection included a shift in its cellular localization pattern within spinal cord neurons. Comparison of spinal cord neurons in animals with k/c-induced arthritis at 4h with neurons in naive animals (Figure 3A), identified markedly increased stain density, especially in the perinuclear regions and in the nucleus (Figure 3B). Similar staining was revealed with either C- or N-terminal NR1 specific antibodies. Pretreatment of animals with genistein effectively abrogated the increased nuclear staining density of NMDA NR1 in spinal neurons of k/c treated animals. The cell shown in Figure 3C was from the spinal segment near the site of genistein administration in a k/c arthritic animal and thus was nearly devoid of NR1 staining.

#### NMDA NR1 Subcellular Localization by Electron Microscopy (EM)

**FIGURE 4:**
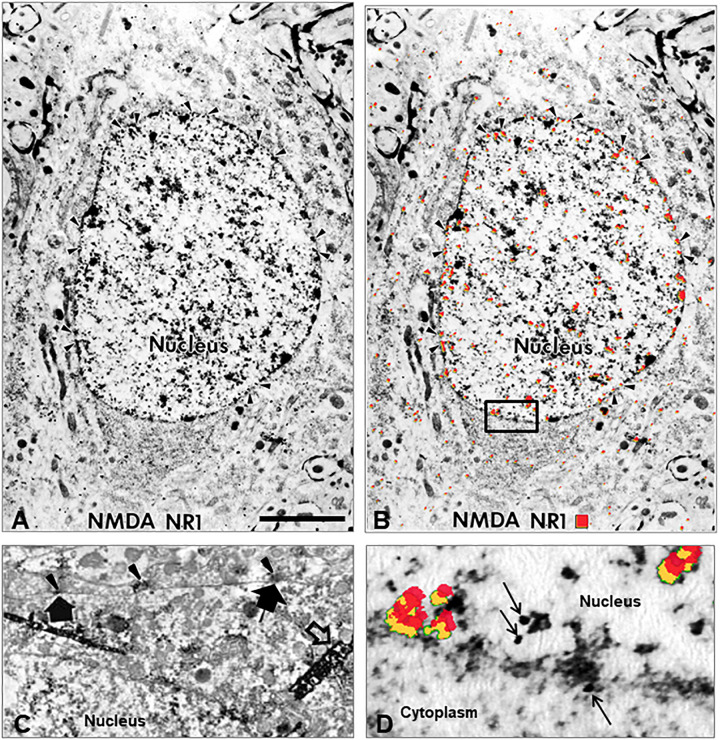
Nuclear translocation of NMDA NR1. **(A)** Electron micrograph (EM) illustrating the intracellular distribution of NMDA glutamate receptor NR1 subunit in a spinal cord neuron 4 h after k/c induction of knee joint inflammation. The immunolocalization of the NR1 subunit is evident in the nucleus, particularly in a distribution pattern reminiscent of other receptor localizations, i.e., at the rim of nuclear pores on the nuclear membrane rather than at the post-synaptic localization typical in spinal neurons of naive rats. **(B)** The same EM image with red pseudocolor enhances the ability to observe the subcellular immunostaining of the colloidal gold particles found particularly in the nucleus, nuclear rim, and nuclear pores (arrowheads). **(C)** NMDA NR1 in naïve animals is typically localized along the cell membrane. The arrowheads indicate NMDA NR1 in the pre-synaptic region of terminals and the large arrows indicate post-synaptic membrane immunolabeling with diaminobenzidine on a spinothalamic tract neuron identified by large dense crystals (open arrows) after WGA-HRP retrograde transport from thalamus (Ye and Westlund, 1996). **(D)** High power EM of the inset outlined in panel **(B)**, with red pseudocolor (yellow shadowing) or arrows to indicate immunogold labeling of NMDA NR1 at the nuclear membrane. (*n* = 3) Scale Bar in A = 3 μm in panels **(A,B)**; 1.33 μm in panel **(C)**; 0.3 μm in panel **(D)**.

Electron microscopy images confirmed NMDA NR1 localization in the nucleus of neuronal cells from the ipsilateral spinal cord of animals with k/c induced arthritis at 4 h (Figure 4). Immunogold labeling was found along the nuclear membrane (Figures 4A,B arrowheads). In naïve animals, immunoperoxidase labeling for NMDA NR1 was primarily localized at synaptic densities on the outer cell membrane (Figure 4C, large arrows) of spinothalamic tract neurons (Figure 4C, large dense crystals; open arrow). For better EM visualization of NMDA NR1 immunolocalization at the synaptic density in a control animal without dense counterstain, refer to Figure 13a in Petralia et al. (1994). The Figure 4D is the higher power image of the nuclear membrane marked as the inset in Figure 4B. Colloidal gold labeled NMDA NR1 was observed both in the cytoplasm and nucleus, including in proximity to the nuclear pore sites (arrows, uncolorized and colorized views). The EM images support nuclear translocation of the NR1 subunit at 4 h that is temporally concurrent to the development of the physiologic and nociceptive arthritic changes.

### Partial Inhibition of NMDA NR1 by a Protein Synthesis Inhibitor

**FIGURE 5:**
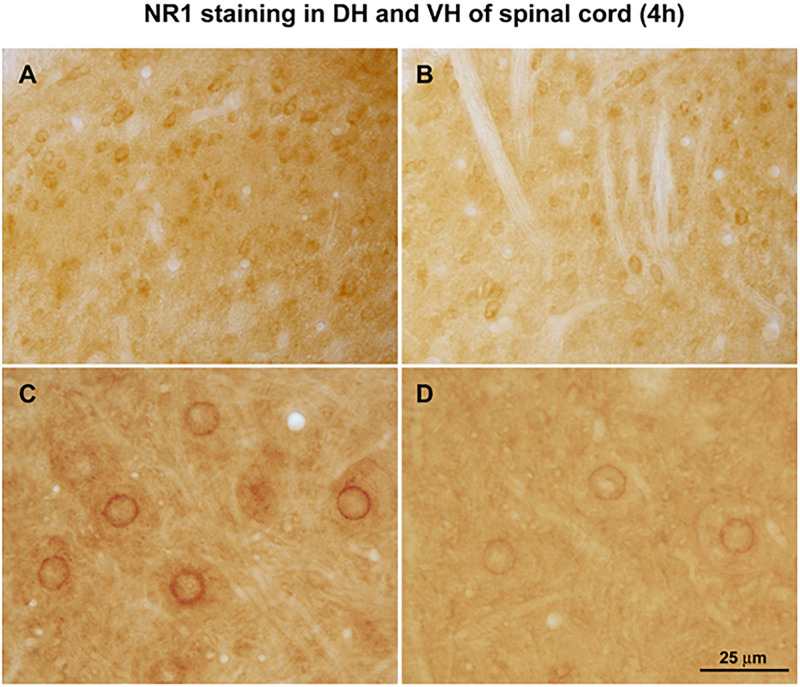
Effect of cycloheximide protein synthesis inhibition. Arthritic rats were pretreated with saline (**A**, dorsal horn and **C**, ventral horn) or protein synthesis inhibitor cycloheximide (30 mg/kg, i.p.) **(B,D)**. Relative increases in NMDA NR1 receptor nuclear rim immunostaining density in arthritic rats could potentially be attributed to newly synthesized protein. Some constitutive NR1 remains. (*n* = 3) Scale Bar = 75 μm in panels **(A,B)** and 25 μm in panels **(C,D)**.

In some animals, pre-treatment with cycloheximide was given before k/c induction of arthritis to eliminate *de novo* protein synthesis. Dorsal horn (Figures 5A,B) and ventral horn sections (Figures 5C,D) from arthritic animals are shown after induction of k/c arthritis. The prominent nuclear rim staining is best appreciated in the ventral horn (Figure 5C). After intraperitoneal pretreatment with either saline (Figures 5A,C) or cycloheximide (30 mg/kg, Figures 5B,D), the increase in spinal cord NR1 staining in arthritic animals was visibly ∼50% less with cycloheximide pretreatment. The remaining nuclear staining present in the cyclohexamide treated arthritic animals indicated NR1 translocation to the nucleus was independent of *de novo* protein synthesis. Pretreatment with transcription inhibitor actinomycin D reduced some of the diffuse staining but not the nuclear translocation. Pre-treatment with leptomycin, a potent, specific nuclear export inhibitor and class II histone deacetylase inhibitor, also reduced nuclear NR1 immunostaining in animals with arthritis. These findings together suggest some of the localization at the nucleus was *de novo* synthesis and some was from intracellular stores.

### Cellular Response to Glutamate on SH-SY5Y Neuroblastoma Cells *in vitro*

#### Increased Expression of NMDA NR1 Subunit and Nuclear Translocation in Human SH-SY5Y Neuroblastoma Cells After Glutamate and NMDA Activation

**FIGURE 6:**
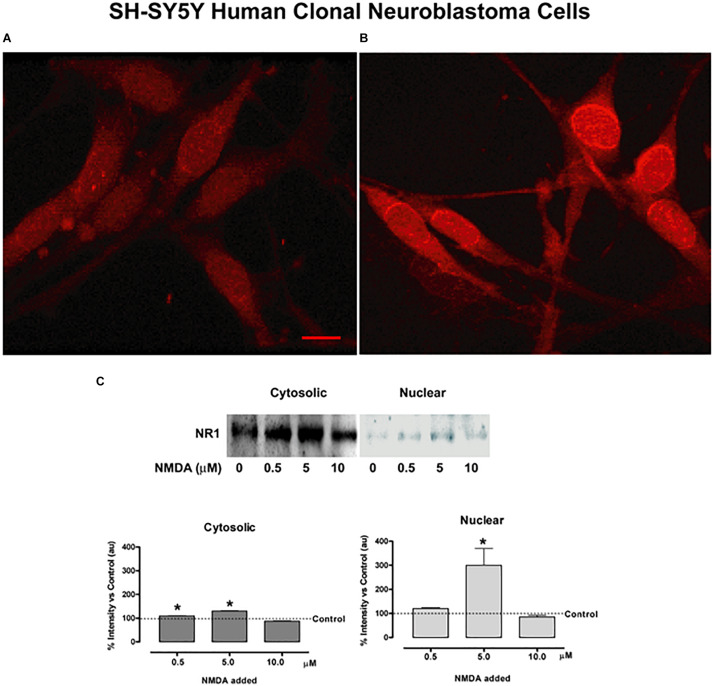
NMDA NR1 staining and nuclear translocation in cultured SH-SY5Y human neuroblastoma cells by glutamate agonists. **(A)** Glutamate NMDA NR1 receptor staining of cultured SH-SY5Y neuroblastoma cells in unstimulated cultures. **(B)** Cells stimulated with 100 μM L-glutamate for 4 h demonstrated increased cytoplasmic and nuclear staining for NR1 compared to control. **(C)** Bands representing one of three studies using Western blot analyses in which cultured SH-SY5Y neuroblastoma cells were stimulated with glutamate receptor agonist NMDA at 0, 0.5, 5.0, or 10.0 μM concentrations for 4 h. NMDA NR1 subunit was detected predominantly in the cytosolic fractions at low NMDA doses and showed increased expression in the nuclear fractions at 5 μM NMDA doses. The bar graphs demonstrate the mean ± SE corrected band densities of cytosolic and nuclear fractions from the three studies performed. Each condition was done in triplicate and repeated 3 times. Scale Bar = 10 μm in panels **(A,B)**. *p < 0.05 compared to unstimulated controls.

Studies performed in clonal SH-SY5Y human neuroblastoma cells further assessed cellular localization of NMDA NR1 with glutamate receptor activation as shown with immunocytochemical studies (Figure 6). Immunocytochemical staining patterns of NMDA NR1 in SH-SY5Y cells treated with 100 μM glutamate for 4 h were compared to unstimulated cells (Figure 6A). Increased intensity of NMDA NR1 staining was observed, with an intracellular shift to perinuclear regions, the nuclear rim, and within the nucleus (Figure 6B). Similar staining was revealed with either C- or N-terminal specific antibodies.

#### Response to NMDA With Western Blot for Cytosolic and Nuclear Expression

Figure 6C demonstrates the same shift in cellular protein localization of NR1 with Western blots in the clonal SH-SY5Y human neuroblastoma cells after treatment with NMDA. Cells were treated with a dilution series of NMDA (0−10 μM), then extracted for cytosolic and nuclear fractions. The NMDA NR1 band densities were evident from the cytosolic fractions in the unstimulated and stimulated cells with added NMDA to a final concentration of 0, 0.5, 5.0 or 10.0 μM NMDA. A smaller but significant mean increase was also noted in the cytosolic fractions at NMDA concentrations of 0.5 and 5 μM compared to the untreated cells (110 ± 0.88, *p* = 0.03 and 130 ± 1.09, *p* = 0.015 vs. 100%, respectively).

Minimal or no NR1 band densities were appreciated in the nuclear fractions above the untreated control cells at NMDA concentrations of 0, 0.1, 0.5, or 1.0 μM, respectively (Figure 6C). However, at 5 μM NMDA, there was a marked increase in NMDA NR1 band density in the nuclear fraction compared to the control cells (300 ± 70 vs. 100%, respectively, *p* = 0.003), indicating a shift to NR1 nuclear localization. As an additional control, human clonal SW982 synoviocytes were treated with NMDA (5 μM) and ACPD (5 μM), and their nuclei isolated. The isolated nuclei had visibly increased NMDA NR1 immunostaining compared to nuclei of untreated cultures (Supplementary Figure S2).

#### Response to Glutamate After Pretreatment With Genistein or Staurosporine

**FIGURE 7:**
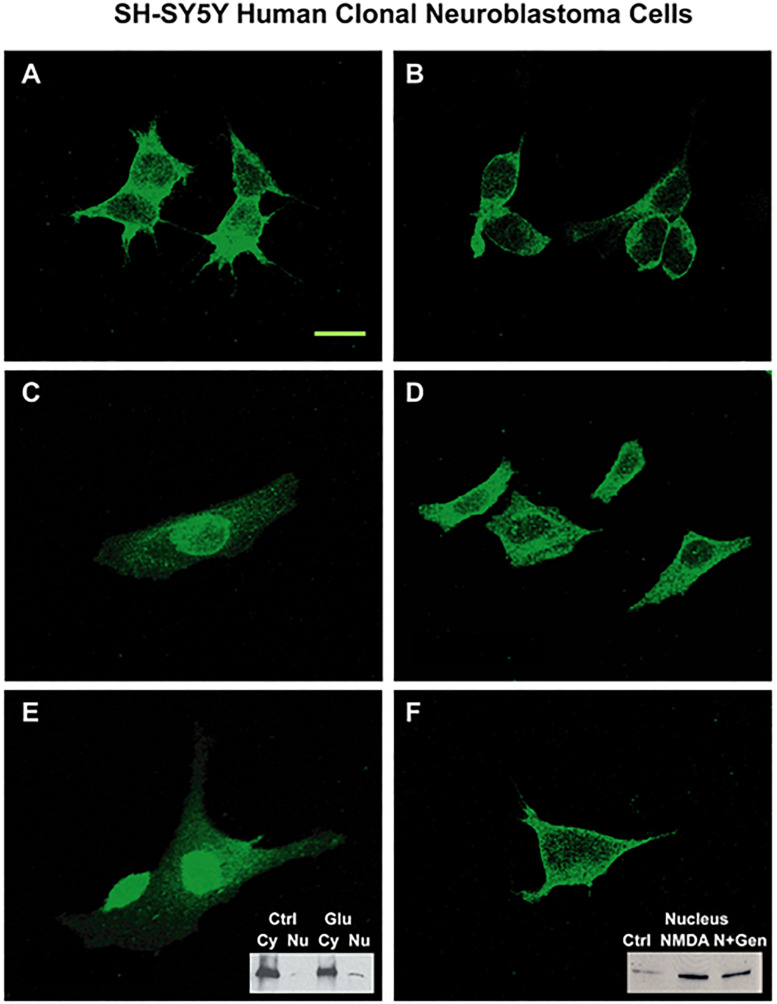
SH-SY5Y neuroblastoma cells activated by glutamate agonists: NMDA NR1 staining and nuclear translocation blunted by PTK inhibitor pre-treatment. **(A)** NMDA NR1 staining of cultured SH-SY5Y neuroblastoma cells in untreated cells. **(C,E)** In comparison, nuclear translocation is noted in cells stimulated with L-glutamate (100 μM) at 4 h; **(B,D)** Cells pretreated with staurosporin (100 nM), a broad spectrum kinase inhibitor, prior to stimulation with L- glutamate do not have nuclear localization. **(F)** Cells pretreated with genistein (50 μM) then stimulated with L-glutamate also do not have nuclear localization. Two insets are provided to demonstrate expression of NR1 in treated cells by Western blot analysis. (**E** inset) Treatment of the cells with L-glutamate increased the expression of NR1 in the nuclear fraction by three-fold. (**F** inset) Pretreatment of the cells with genistein diminished the expected increase of NR1 seen with treatment of NMDA (5 μM) in the nuclear fraction by 40%. Each condition was done in triplicate and repeated 3 times. Scale Bar = 20 μm.

N-methyl-D-aspartate NR1 nuclear localization was further assessed in clonal SH-SY5Y human neuroblastoma cell cultures pretreated with protein kinase inhibitors, genistein or staurosporin, as shown in Figure 7. Compared to unstimulated cells (Figure 7A), cells incubated with 100 μM glutamate for 4 h (Figures 7C,E) had increased staining in nuclear, nuclear rim, and perinuclear regions. Pretreatment with 50 μM genistein (Figure 7F) or 100 nM staurosporine (Figures 7B,D) before glutamate incubation resulted in minimal nuclear staining of NMDA NR1. Staining at the cell border was intense, and staining in the perinuclear region was still evident. Two insets are provided to demonstrate semi-quantitative Western blot analysis of NR1 expression in treated cells. Treatment of the cells with 100 μM L-glutamate increased the expression of NR1 in the nuclear fraction 3-fold (Figure 7E inset). Pretreatment of the cells with 50 μM genistein diminished the expected increase of NR1 seen with treatment of 5 μM NMDA in the nuclear fraction by 40% (Figure 7F inset).

## Discussion

In an acute arthritis model, we provided evidence that NMDA NR1 subunit is increased and showed cellular redistribution that temporally corresponded with secondary thermal hypersensitivity. Direct delivery of PTK inhibitors genistein or lavendustin A to the affected spinal cord segment by microdialysis blocked both the increase in cellular NMDA NR1 expression and its subcellular redistribution. Importantly, lumbar pretreatment with these PTK inhibitors resulted in significant blunting of the secondary hyperalgesic response in a dose dependent manner, indicated by increased paw withdrawal latency at 4 h compared to vehicle treatment. However, this *spinal* administration did not impact scores of joint circumference or surface temperature at 4 hr. Similarly, the *in vitro* studies in clonal neuronal cell cultures activated with glutamate reflected similar increases in NMDA NR1 protein expression and subcellular redistribution that were blunted with PTK inhibitors.

The early increases in the NMDA NR1 subunit by 4 h and the shift to the nuclear compartment shown here with an *in vivo* arthritis model and the *in vitro* studies provide evidence of the importance of NMDA NR1 as an intracellular signaling molecule that likely impacts downstream cellular function directly at the nucleus. The studies with the PTK inhibitors provide evidence that protein tyrosine phosphorylation is important in (i) modulation of nociceptive responses induced by peripheral k/c arthritis, (ii) induced NMDA NR1 subunit expression increases in the spinal cord, and (iii) NMDA NR1 subunit trafficking from the cell membrane to the nuclear membrane. The present studies add to proposed mechanisms whereby non-receptor tyrosine kinase phosphorylation events alter neuronal responsivity with resultant nociceptive hypersensitivity (Obreja et al., 2002). These data support direct involvement of NR1 nuclear translocation in modification of transcriptional responses to glutamate.

### The Role of PTK in the Development of Secondary Hyperalgesia

The PTK inhibitors have been suggested as effective therapeutic interventions for inflammatory pain (Bonilla-Hernán et al., 2011). Our current study and previous studies by others support the hypothesis that NMDA activation initiates tyrosine kinase mediated events that generate hyperalgesic behavioral responses (Obreja et al., 2002; Zhu et al., 2012). Persistent enhancement of glutamate release in spinal cord contributes to altered neuronal responsiveness precipitating hypersensitivity. The intra-articular k/c model utilized here inflamed the knee joint allowing nociceptive testing of the ipsilateral uninjured footpad and a clear secondary heat hypersensitivity response. This model of acute joint injury was used to demonstrate the onset of acute nociceptive changes that will occur with joint trauma, and how this can be blunted with one temporally related pretreatment dosing of a PTK inhibitor such as genistein.

Spinal cord increase in NMDA NR1 protein and its nuclear translocation within 4 h after k/c arthritis induction are coincident in time with development of peripheral inflammation and secondary hyperalgesia. Tyrosine phosphorylation of the NR2B, but not the NR2A, is also associated with the development of persistent pain after inflammation (Guo et al., 2002). Our previous studies have supported a pivotal role for spinal cord excitatory amino acid release and its role in activation of spinothalamic pain pathway neurons and central sensitization (Dougherty et al., 1992a, b; Sorkin et al., 1992; Sluka and Westlund, 1993a). Others have demonstrated subsequent activation of NMDA NR1 in the dorsal horn initiated by peripheral injury, including arthritis (reviewed in Dubner and Ruda, 1992; Woolf and Costigan, 1999; Guo et al., 2002; South et al., 2003). Alternatively, depletion of NMDA NR1 in the dorsal horn or its targeted blockade with use of NMDA antagonists or antisense mRNA produces a spectrum of response blunting, through attenuation of injury- initiated responses (Sluka and Westlund, 1992, 1993b; Woolf and Salter, 2000; South et al., 2003; Inturrisi, 2005).

### Arthritis-Induced Spinal Cord NMDA NR1 Elevations Are Blocked by Pretreatment With Genistein

These NMDA mediated events in the spinal cord facilitate neuronal plasticity events and ultimately induce complex inflammatory *and* neural contributors to central sensitization The NMDA NR1 increases in the spinal cord are consistent with long-term potentiation in other parts of the neuraxis, such as the hippocampus (Abe and Saito, 1993; Bartanusz et al., 1995; Yu et al., 1997). This involves phosphorylation of NR1 and NR2B subunits and can be prevented by activation and interactions with G protein-coupled mGluR and NK1 tachykinin receptors that increase intracellular Ca^2 +^ (Guo et al., 2002; Zhang et al., 2002; Caudle et al., 2005). Previous *in vitro* studies have shown that genistein inhibits nociceptive trigeminal neuron excitability through a non-specific inhibition of voltage dependent sodium channels (Liu et al., 2004). Selective deletion of 80% of NMDA NR1 subunit in lumbar spinal cord by injection of adeno-associated virus expressing Cre recombinase into floxed NR1 mice results in functional loss of NMDA, but not AMPA currents (Inturrisi, 2005). In contrast to the present study, phosphorylated NR1 has been shown to be increased in spinal cord after brief intense activation of peripheral afferent nerves with capsaicin and in the streptozotocin induced rodent diabetes model (Zou et al., 2002; Daulhac et al., 2011; Wang et al., 2017). The visibly reduced spinal cord phosphoNR1 in a chronic neuropathic pain model may reflect long term depletion (Supplementary Figure S1). Less phosphoNR1 in Lamina I and II in the arthritic animals at 4 h may reflect maximal utilization (Figure 3). Intense afferent activation would result in increased new translational expression of NMDA NR1 and membrane insertion from intracellular stores by 4 h. Meanwhile, phosphoNR1 utilization would be maximal. In any event the data here is quite different and may simply reflect antibody binding to different phosphoNR1 variants.

### Nuclear Localization of NMDA NR1

Nuclear translocation of the NMDA NR1 subunit was evident by light and electron microscopy in both dorsal and ventral horn neurons and in glial cells using both N- and C-terminal antibodies directed against NMDA NR1, indicating that the NR1 subunit was intact.

The NMDA NR1 subunit has a putative nuclear localization signal (NLS) region required for this translocation (Acc Number NM_007327 Human NMDA NR1 nucleotide sequence, GenBank, NCBI). Holmes et al. (2002a,b) have reported functional NLS sequences for NR1-1 and NR1-4 splice variants located equally in both the cytoplasm and nucleus, suggesting their small size allows ready passage through nuclear pores (Holmes et al., 2002a,b). Involvement of released serine and the inter-organelle signaling modulator/chaperone, Sigma-1 receptor, are proposed as functional mediators in NMDA NR1 upregulation, translocation, phosphorylation, central sensitization, and nociceptive hypersensitivity (Sluka and Westlund, 1993a,b; Zou et al., 2000; Kim et al., 2008; Roh et al., 2008; Su et al., 2010; Azkona et al., 2016; Choi et al., 2018). The EM photographs provided here support an easy passage through nuclear pores for NMDA NR1 (Figures 4A,B,D).

Subcellular localization in the nucleus has been characterized for transfected cell cultures in molecular studies (Marsh et al., 2001; Holmes et al., 2002a, b). In the k/c inflammatory arthritis model, NMDA NR1 was the only glutamate receptor subunit of those tested whose expression significantly increased in the gray matter, although NR2 upregulation has been reported in more intense formalin-induced and CFA inflammation models (Gaunitz et al., 2002; Azkona et al., 2016). NMDA NR1 localization in control animals more typically is shown with EM to represent post-synaptic membrane localization (Liu et al., 1994; Petralia et al., 1994; Ye et al., 1999). Previous reports have also implicated NR1 cellular membrane associated translocations for subunit recycling.

The numerous present studies support nuclear translocation as one responsibility for the glutamate NMDA NR1-1 not shared by other glutamate NR1-4 subunits in initial hours after persisting afferent nerve activation, though it is not clear at this point which of the glutamate NR1-1 and NR1-4 subunits might be responsible.

The nuclear translocation of the NR1 subunit after glutamate activation suggests NR1 protein also plays a direct role in the fast intracellular signaling responses to glutamate activation. The histological data demonstrating a shift in the subcellular location of the NR1 subunit supports NR1’s role as a rapid intracellular mediator acting through direct communication with the nucleus. The findings indicate activated NMDA could produce rapid downstream events resulting in increased NR1 subunit protein potentially required to mobilize the shift in NR2 subunit composition and numbers that promote hypersensitivity in this acute arthritis model.

The modulation of trafficking to the cell nucleus promoting transcriptional changes that impact NMDA ion channel function impacts the well characterized mechanisms generating LTP that extends activation for days and weeks in the hippocampus (Abe and Saito, 1993; Wang and Salter, 1994; Fadool et al., 1997; Yu et al., 1997; Lu et al., 1998). Activation of NMDA NR1 signaling pathway is correlated with miRNA219 levels, long-term potentiation (LTP), and hippocampal pyramidal cell number (Zhang et al., 2019).

Activation of membrane associated NMDA channels modulates trafficking of signaling mediators to the cell nucleus, such a MAPK, NFκB, PKA, NFAT and calmodulin (reviewed in Thompson et al., 2004). Activity dependent binding and phosphorylation of importin α to the NLS present in the cytoplasmic tail of NR1-1a provides translocation from the synapse to the nucleus during transcription-dependent forms of neuronal plasticity (Jeffrey et al., 2009). Diversity of NMDA receptor configurations providing a variety of resultant physiological and pharmacological properties and functions is based on differential RNA splicing (Zukin and Bennett, 1995). Differing results in expression after injury is reported depending on timing after injury, location of the injury, and the splice variant recognized by the particular antibodies utilized (Zou et al., 2000, 2002; Gaunitz et al., 2002; Guo et al., 2002; Caudle et al., 2003, 2005; Brenner et al., 2004). The persisting hypothesis is that alternatively spliced cassettes of the NR1 protein play differing roles in the functional characteristics of the NMDA receptors adjusting to specific physiological or injury states (Prybylowski et al., 2001). It is clear that the timing of migration to the nucleus is rapid and coincides with the development of hypersensitivity.

Central sensitization, however, is a highly complex and interactive process involving numerous neurotransmitter events. Phosphorylation of NMDA NR1 after intradermal capsaicin injection is increased at 30 and 60 min in the spinal cord dorsal horn (Zou et al., 2000). In that study, there was no difference from naive at subsequent 120 and 180 min time points as hypersensitivity resolved. Other studies have also found that phosphoNR1 is increased after temporomandibular joint inflammation is induced with carrageenan (Cavalcante et al., 2013). The present data indicate that at 4 h, there is decreased staining density in lumbar spinal cord phosphoNR1 in animals with k/c arthritis compared to naïve animals. Likewise, in a chronic trigeminal neuropathic pain model with persisting hypersensitivity, staining density for phosphoNR1 in C1 spinal cord was less than in naïve animals at 10 weeks (see Supplementary Figure S1). The significance of this variability in different pain models captured at various time points is not entirely known. Likely, variability is due to the timing of different molecular signaling mechanisms along with rapid versus slow gearing up and also likely entailing depletions with long term persistence. The present study supports NMDA NR1 presence in the nucleus corresponding to molecular events occurring in the initial 2−4 h after intense knee joint afferent activation and central sensitization resulting in secondary hypersensitivity on the footpad. Taken together, considering many other studies the timing of molecular signaling events producing hypersensitivity depends on whether the study is done in the acute or chronic pain setting, which model is studied, what time point is selected (hours, days, months), and the nature of the stimulus.

The lack of effect on swelling of the isolated joint space itself in the present study is not surprising. We have previously shown a spinal NMDA antagonist does not reduce joint swelling, while a non-NMDA antagonist does reduce joint swelling (Sluka et al., 1994). Numerous studies have reported inflammatory cytokines in synovial fluids obtained from knees after direct trauma (Hooshmand et al., 2007; Liu et al., 2019). Elevated levels of synovial fluid excitatory amino acids glutamate (Glu) and aspartate (Asp) from traumatic joints in humans have been reported (McNearney et al., 2000). The addition of NMDA has been shown to increase NMDA NR1 and inflammatory cytokines in primary and clonal human synoviocyte cultures (Flood et al., 2007; McNearney et al., 2010). NMDA and ACPD induced increase in tumor necrosis factor alpha (TNFα) is also blunted by genistein pretreatment (Supplementary Figure S3). Other reports have found physiologic relevance to glutamate and NMDA receptors and elevated glutamate levels in other peripheral tissues, including bone (Chenu et al., 1998; Gu and Publicover, 2000; Gu et al., 2002) and tendons (Kader et al., 2002; Svedman et al., 2018). Knee joint glutamate increases have been reported in patients with Achilles tendinitis (Alfredson and Lorentzon, 2003).

### Block of NMDA NR1 Nuclear Localization With Genistein

Transport of NR1 subunit to the nucleus was markedly reduced after pretreatment with various tyrosine kinase inhibitors in both spinal cord and clonal neuroblastoma cell cultures, indicating the importance of tyrosine kinase phosphorylation events to cellular transport in injury responses. The concentration of genistein used (50 μM) can also directly block glutamate activation of NMDA channels (Huang et al., 2010). Genistein does not inhibit all nuclear translocation events (Htun et al., 1998), but anti- inflammatory effects are reportedly efficacious in disorders related to steroid receptors (Garcia Palacios et al., 2005) and especially protein kinase mediated cell signaling events involved in translocation. Inhibition of protein synthesis by pretreatment with cycloheximide reduced neuronal NR1 staining by about 50% in spinal neurons but did not abrogate all nuclear staining. Thus, nuclear translocation of constitutive NR1 occurs after glutamate activation prior to *de novo* synthesis of NMDA NR1. In a previous *in vitro* study assessing the consequence of NMDA NR1 activation in human cultured synoviocytes, we reported that nuclear translocation after NMDA addition is evident within 1−2 h (McNearney et al., 2010). In that study, nuclear translocation of NMDA NR1 in activated synoviocytes was blocked by pre-treatment with MK-801.

### Genistein Improves Viability and Has Neuroprotective Effects

Emerging clinical studies are reporting improvements in pain, systemic inflammatory, and cancer symptoms with genistein treatment (Gupta et al., 2011; Liu et al., 2019). Genistein inhibits reactive oxygen species production and aggregation of platelets in rats (Schoene and Guidry, 1999). Other studies report the peripheral use of genistein, related agents and isoflavones have local anti-inflammatory activity (by PTK inhibition) *in vitro* and in animal models, including arthritis (Orlicek et al., 1999; Verdrengh et al., 2003; Dijsselbloem et al., 2004; McNearney et al., 2010; Mohammad-Shahi et al., 2011; Li et al., 2014; Liu et al., 2019). For example, genistein blocks thermal hypersensitivity in inflammatory pain models (Lu et al., 2000; Guo et al., 2002). Genistein reduced serum granulocyte, monocyte, and lymphocyte inflammatory response to collagen-induced arthritis as either pretreatment or post- treatment (Verdrengh et al., 2003). In addition to its ability to inhibit PTK, genistein blocks NMDA receptors via a rapid, voltage-dependent, PTK-independent mode of action at an extracellular site (Huang et al., 2010). In that study, Lavendustin A is a specific PTK inhibitor analog with no direct effect on NMDA activated current. With continuous subcutaneous administration of genistein, reductions in sciatic nerve expression of NF-κB, IL-1β and IL-6 mRNAs, neuronal and inducible nitric oxide synthase in DRG, spinal cord and thalamus were reported in a sciatic nerve ligation model (Valsecchi et al., 2008). Genistein also reduced NGF, oxidative stress, inflammation, and nociceptive hypersensitivity in a time- and dose-dependent manner, and vascular dysfunction that follow streptozotocin induced diabetes in mice (Valsecchi et al., 2011). Genistein, but not inactive daidzein, reduced the rise in serum TNF alpha levels caused by systemic LPS (Ruetten and Thiemermann, 1997) (see also Supplementary Figure S3).

Genistein’s neuroprotective effects were demonstrated *in vitro* in neurodegeneration models with both pre- and post-treatment protocols. Pre-treated PC-12 or post-treated SH-SY5Y cells exposed to β-amyloid or β-amyloid fragment (Aβ_25__–__35_) are protected from apoptotic events (Tan and Kim, 2016; Xu et al., 2019). Genistein post-treatment reduces Bax mRNA increase and Bcl-2 mRNA expression decrease to Aβ_25__–__35_ exposure (Xu et al., 2019). Aβ_25__–__35_ induced increases in Ca^2 +^ concentration decreased in response to genistein shown with intracellular fluorescent dye indicator response (Xu et al., 2019). Genistein increased expression of the GluR2 and decreased NR2B subunit (Guo et al., 2002; Xu et al., 2019). A commonality in these effects may be related to genistein’s reported ability to directly block glutamate activation of NMDA channels (Huang et al., 2010).

### Limitations of the Study

Limitations noted in the study and considerations of the related literature include the necessity to determine the dynamics of PTK phosphorylation in other inflammatory models. Differences here compared to other literature reports appear to be related more to the nature of the models under study, mechanisms involved in different insults whether inflammatory or neurogenic, and the timing involved for development of insult response. Likewise, consideration of whether the treatments are pre- or post- model induction is necessary to establish the relationship of inflammatory or neurogenic events to secondary hypersensitivity and the mechanisms that might limit central sensitization. Our study is limited to one time point, during maximal arthritic injury in an acute arthritis model. A time course might be necessary to assess the timing and dosing of PTK inhibitors in arthritis models, to identify the protocol that reflects minimal joint injury or trauma. Another consideration is to question if systemic, intra-articular or oral ingestion of genistein would best mitigate secondary hypersensitivity. Or would systemic administration be more efficacious for extra-articular structures of the joint. Since many variations and models are examined, our study with direct spinal administration is but one to consider.

## Conclusion

The development of secondary thermal hyperalgesia and upregulation of NR1 protein in response to peripheral tissue injury requires phosphorylation events mediated by non-receptor, protein tyrosine kinase. The increased expression and shift of the NMDA NR1 subunit to the nuclear compartment occurring in response to induction of arthritis (4 h) is concomitant with the development of spinal level sensitization indicated by secondary thermal hyperalgesia. Combined with the *in vitro* data, we have provided evidence that glutamate NMDA NR1 subunit plays a pivotal role as a rapidly responsive intracellular signaling mediator of NMDA-mediated glutamate activation mediated by protein tyrosine kinase. This pretreatment protocol with a PTK inhibitor that blunts neuronal NMDA NR1 increases, and translocation initiated changes has generated speculation that currently available PTK inhibitors, such as genistein, might offer clinical efficacy with pretreatment, “on-demand” dosing. Future studies will determine if this can be translated to a preemptive or prophylactic dosing strategy to benefit from the analgesic and possibly chondroprotective functions of PTK inhibitors in the immediate phase of acute joint trauma in humans, as shown here in a rat arthritis model.

## Data Availability Statement

The datasets generated for this study are available on request to the corresponding author.

## Ethics Statement

The animal studies were reviewed and approved by the University of Texas Medical Center Galveston IACUC and the New Mexico Veterans Administration Health Care System (NMVAHCS) IACUC review committees.

## Author Contributions

KW, TM, and TP conceived the study and designed the experiments. YL, W-RZ, GT, TP, SM, and LZ performed the experiments. TM, YL, TP, and LZ analyzed the data. KW and TM wrote the manuscript. All authors read and approved the final version of the manuscript.

## Conflict of Interest

TP was currently employed by the Everlywell Inc., Austin, TX, United States though not at the time that these studies were performed. The remaining authors declare that the research was conducted in the absence of any commercial or financial relationships that could be construed as a potential conflict of interest.

## Supplementary Material

FIGURE S1 Expression of phosphorylated NR1 subunit is decreased in a chronic pain model. Expression of phosphorylated NR1 (pNR1) subunit is decreased in a model of chronic trigeminal neuropathic pain (CCI-ION) in rat cervical spinal cord. Immunostaining for pNR1 (green) in cervical spinal cord section from naïve control rat **(A)** and 12 weeks after unilateral chronic constriction injury of the infraorbital branch of the trigeminal nerve **(B)** was more intense in the uninjured sample. Tissue samples were counter stained with DAPI (blue) (*n* = 3).

FIGURE S2 NMDA NR1 in isolated SW892 human clonal synoviocyte nuclei. Nuclei isolated from treated and untreated cultures of SW892 human clonal synoviocytes were immunostained for NMDA NR1. Cultures were treated with NMDA and ACPD (5 μM each, 12 h) the nuclei were isolated from cytoplasm and FAC sorted. Three replicates minimum were studied. Scale Bar = 50 μm **A.** NMDA NR1 staining of untreated isolated SW892 human clonal synoviocyte nuclei **B.** NMDANR1 staining of isolated SW892 human clonal synoviocyte nuclei from cell cultures treated with NMDA + ACPD.

FIGURE S3 NMDA and ACPD stimulated TNF-alpha release from SW892 human clonal synoviocytes is blunted following preincubation with PTK inhibitor genistein. Human clonal SW892 synovial cells were cultured to 2×106 million cells/well. Cell were pre-incubated with genistein 5 μM, or vehicle for 1 hour at 37°C. After 1 hour, 5 μM NMDA and 5 μM ACPD or vehicle was added to assigned wells. The plates were incubated at 37°*C* for 24 hours. At 24 hours, cell culture supernatant was harvested from each well and TNF-alpha levels were ascertained by immunoassay (R&D Systems, MN, USA). Cells were also evaluated for viability with trypan blue exclusion and LDH release. Each condition was done in triplicate and repeated 3 times. This graph demonstrates low levels of supernatant TNF-alpha in untreated cells. When cells were incubated with NMDA and ACPD, there was a robust increase in supernatant TNF-alpha. Cells incubated with genistein alone showed a slight increase over baseline levels seen in the untreated cells. Cells preincubated with genistein before additions of NMDA and ACPD demonstrated a marked decrease in supernatant TNF alpha at 24 hours. This figure demonstrates that genistein incubation with synoviocyte cultures can impact cytokine response to neurotransmitter stimulation. Genistein has been reported to blunt arthritic response in other experimental studies (Mohammad-Shahi et al., 2011; Li et al., 2014; Liu et al., 2019).

## Abbreviations

aCSF: artificial cerebrospinal fluid
DAB: diaminobenzidine
DRG: dorsal root ganglia
EM: electron microscopy
GABA: gamma amino butyric acid
k/c: kaolin/carrageenan
LPS: lipopolysaccharide
NGF: nerve growth factor
NMDA: N-methyl -D-aspartate
NR1: NMDA receptor 1
PTK: non-receptor protein tyrosine kinase
PWL: paw withdrawal latency
WGA-HRP: wheat germ agglutinin-horse radish peroxidase.
