# Supplementary figures and images for: Tyrosine Kinase Inhibitors Reduce NMDA NR1 Subunit Expression, Nuclear Translocation, and Behavioral Pain Measures in Experimental Arthritis
Source: Front Physiol. 2020 May 27;11:440. doi: 10.3389/fphys.2020.00440 (PMC7267073; doi:10.3389/fphys.2020.00440)

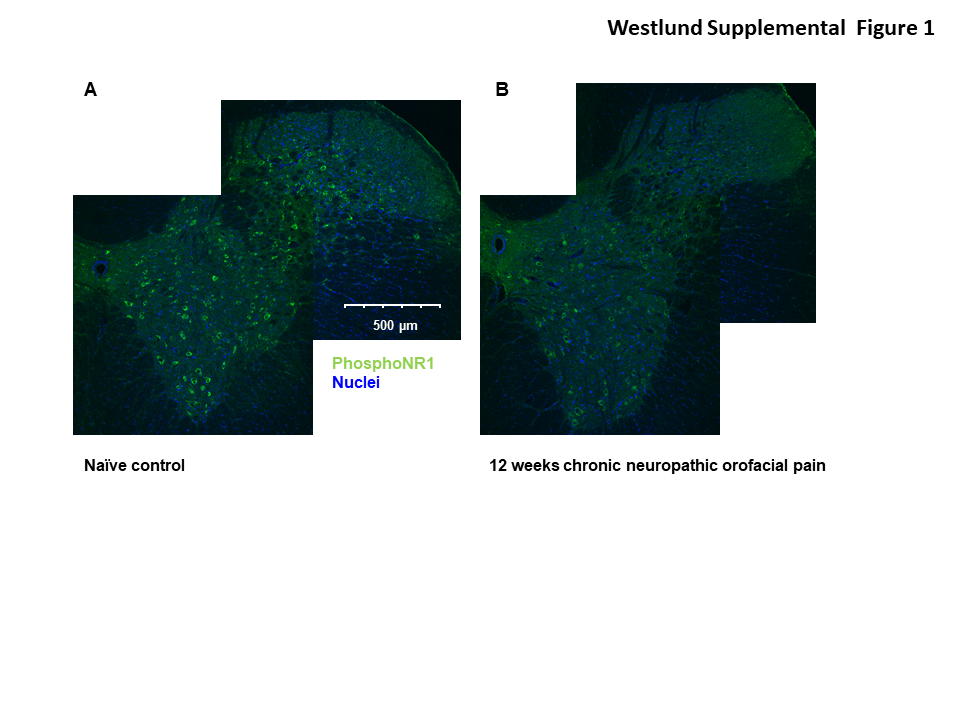

Supplement: FIGURE S1 — Expression of phosphorylated NR1 subunit is decreased in a chronic pain model. Expression of phosphorylated NR1 (pNR1) subunit is decreased in a model of chronic trigeminal neuropathic pain (CCI-ION) in rat cervical spinal cord. Immunostaining for pNR1 (green) in cervical spinal cord section from naïve control rat (A) and 12 weeks after unilateral chronic constriction injury of the infraorbital branch of the trigeminal nerve (B) was more intense in the uninjured sample. Tissue samples were counter stained with DAPI (blue) (n = 3). [file Image_1.TIF]

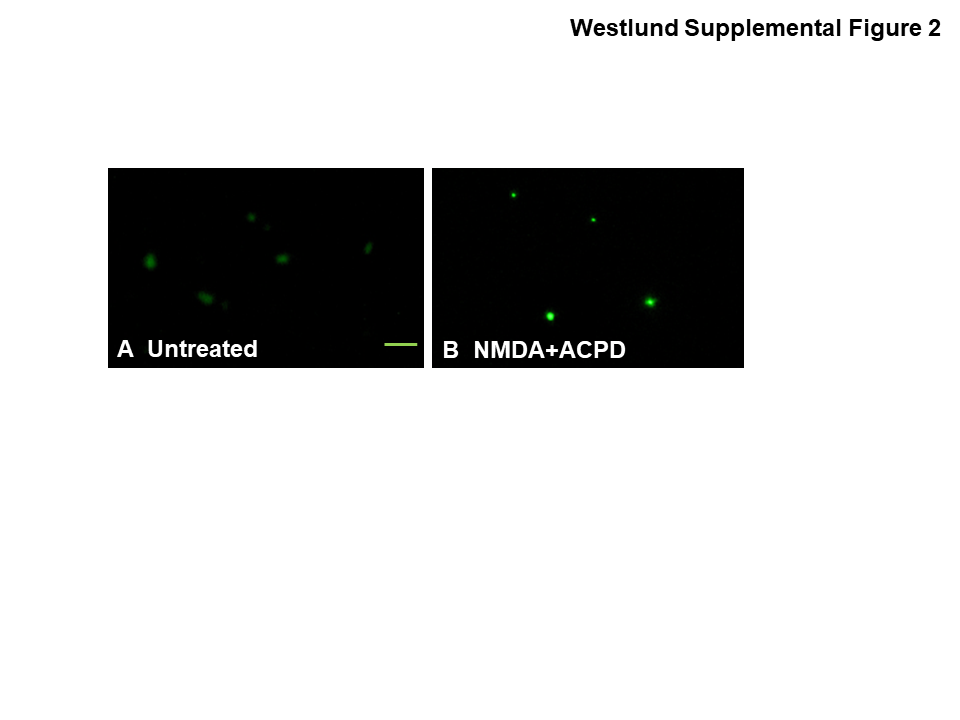

Supplement: FIGURE S2 — NMDA NR1 in isolated SW892 human clonal synoviocyte nuclei. Nuclei isolated from treated and untreated cultures of SW892 human clonal synoviocytes were immunostained for NMDA NR1. Cultures were treated with NMDA and ACPD (5 μM each, 12 h) the nuclei were isolated from cytoplasm and FAC sorted. Three replicates minimum were studied. Scale Bar = 50 μm A. NMDA NR1 staining of untreated isolated SW892 human clonal synoviocyte nuclei B. NMDANR1 staining of isolated SW892 human clonal synoviocyte nuclei from cell cultures treated with NMDA + ACPD. [file Image_2.TIF]

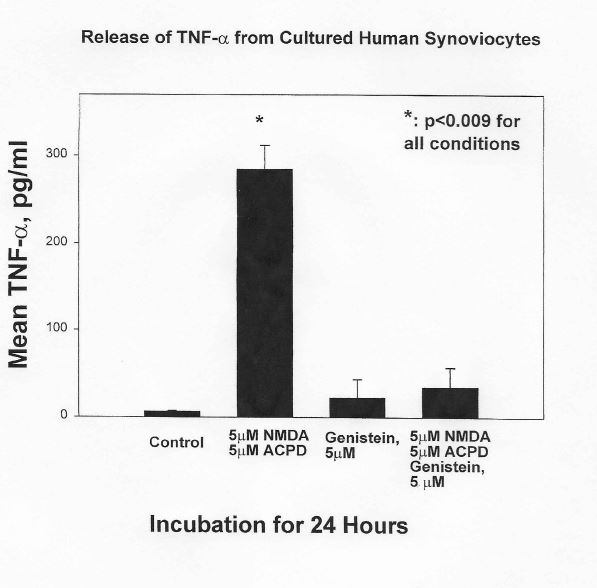

Supplement: FIGURE S3 — NMDA and ACPD stimulated TNF-alpha release from SW892 human clonal synoviocytes is blunted following preincubation with PTK inhibitor genistein. Human clonal SW892 synovial cells were cultured to 2×10^6^ million cells/well. Cell were pre-incubated with genistein 5 μM, or vehicle for 1 hour at 37°C. After 1 hour, 5 μM NMDA and 5 μM ACPD or vehicle was added to assigned wells. The plates were incubated at 37°C for 24 hours. At 24 hours, cell culture supernatant was harvested from each well and TNF-alpha levels were ascertained by immunoassay (R&D Systems, MN, USA). Cells were also evaluated for viability with trypan blue exclusion and LDH release. Each condition was done in triplicate and repeated 3 times. This graph demonstrates low levels of supernatant TNF-alpha in untreated cells. When cells were incubated with NMDA and ACPD, there was a robust increase in supernatant TNF-alpha. Cells incubated with genistein alone showed a slight increase over baseline levels seen in the untreated cells. Cells preincubated with genistein before additions of NMDA and ACPD demonstrated a marked decrease in supernatant TNF alpha at 24 hours. This figure demonstrates that genistein incubation with synoviocyte cultures can impact cytokine response to neurotransmitter stimulation. Genistein has been reported to blunt arthritic response in other experimental studies (Mohammad-Shahi et al., 2011; Li et al., 2014; Liu et al., 2019). [file Image_3.JPEG]
